# Knowledge, attitudes, and practices of *Anopheles* mosquito control through insecticide treated nets and community-based health programs to prevent malaria in East Sumba Island, Indonesia

**DOI:** 10.1371/journal.pgph.0000241

**Published:** 2022-09-02

**Authors:** John T. Bandzuh, Kacey C. Ernst, Jayleen K. L. Gunn, Salmon Pandarangga, Linda Rambu Kuba Yowi, Sarah Hobgen, Kerry R. Cavanaugh, Rambu Yetti Kalaway, Norlina Rambu Jola Kalunga, Maklon Felipus Killa, Umbu Ho Ara, Christopher K. Uejio, Mary H. Hayden

**Affiliations:** 1 Department of Geography, College of Social Science and Public Policy, Florida State University, Tallahassee, FL, United States of America; 2 Department of Epidemiology and Biostatistics, College of Public Health, University of Arizona, Tucson, AZ, United States of America; 3 Universitas Kristen Wira Wacana Sumba, East Sumba Island, Indonesia; 4 Kampung Raja, East Sumba Island, Indonesia; 5 London School of Hygiene and Tropical Medicine, London, United Kingdom; 6 University of Colorado, Lyda Hill Institute for Human Resilience, Colorado Springs, CO, United States of America; McGill University, CANADA

## Abstract

With an estimated 241 million human cases and 627,000 deaths in 2020, malaria remains a significant and ongoing global health challenge. This study employs a qualitative approach to investigate knowledge, attitudes, and practices surrounding mosquito control and prevention methods in East Sumba Regency, Indonesia. While malaria is under control in much of Indonesia, transmission in Sumba Island remains high, with incidence as high as 500 per 1000 population in some areas. A qualitative study was undertaken to explore use of insecticide treated nets, (ITNs), traditional Sumbanese mosquito control methods, and the role of women, integrated health service posts, (*posyandu*) and community-based health workers (*kaders*) in combatting malaria and controlling mosquitoes. Focus group discussions (n = 7) were conducted in East Sumba Island stratified by urban/rural location and level of malaria transmission. Key informant interviews (n = 14) were conducted with religious leaders, health workers, and women’s group leaders. Results indicate that environmental conditions, such as high temperatures, were common deterrents to regular ITN use. Furthermore, our results suggest that community embedded health workers, *kaders*, and health service posts, *posyandu*, play an important role in information dissemination related to mosquitoes and mosquito-borne diseases as well as the distribution and use of ITNs in East Sumba Island. The role of the *posyandu* and *kaders* could be expanded to improve malaria prevention by integration with educational campaigns, aiding ITN distributions, and malaria diagnosis and treatment. This study is the first to examine mosquito-borne disease-related knowledge, attitudes, and practices in East Sumba Island, Indonesia. Results could improve mosquito control and malaria prevention by providing insights into local knowledge of *Anopheles* mosquitoes and malaria as well. Tailoring mosquito control and malaria prevention strategies around local knowledge and perceptions is likely to be more acceptable and sustainable.

## Background

A global effort to combat malaria began in 1955 with the inception of the World Health Organization (WHO) Global Malaria Eradication Programme [1]. Today, the organization works toward malaria elimination and the prevention of re-introduction in areas where elimination has been successful. The WHO South-East Asia region had the largest reduction of malaria incidence globally from 23 million cases in 2010 to 6.3 million cases in 2019 [2]. Despite a reduction of 73%, WHO South-East Asia still ranks second in number of malaria cases, only behind the WHO Africa region. Although malaria remains a major health problem in Indonesia, progress toward elimination was evident with a 50% decline in confirmed cases and a 66% reduction in deaths between 2007 and 2017 [3]. However, from 2016 to 2017, malaria incidence in Indonesia increased [3], and equal numbers of *Plasmodium falciparum* and *Plasmodium vivax* cases were reported [4, 5]. While most of Indonesia (94%) has achieved low levels of transmission (<1 per 1000), 6% of the population still has high transmission, including East Sumba Island [5].

This study centers on East Sumba Island, Indonesia where efforts to combat mosquito-borne diseases including malaria and dengue focus on distribution of ITNs, source reduction, insecticides, and prophylaxis. Use of ITNs has successfully reduced malaria in many other regions of Indonesia; thus, this work explored the use of ITNs in East Sumba and the role of a predominantly women-led community-based health program known as *posyandu* to reduce malaria. Our original research focus was to investigate the opportunities and barriers for a more gender inclusive mosquito control landscape [6]. This led us to predominantly engage women-led organizations and women in data collection.

The Village Community Health (*Pembangungan Kesehatan Masyarakat Desa* or PKMD) was established in Indonesia in 1975 as a community-based system to address health issues for those living in poverty [7]. *Posyandu* (integrated healthcare post) formed in 1984 to expand the ability of PKMD to comprehensively address issues such as family planning, nutrition, immunizations, and other matters. *Posyandu* originally focused on child and maternal health to achieve goals set forth in the Health for All program [8]. However, it has since been expanded to cover additional topics and community needs. A *posyandu* operates as a key rural health care institution and relies on cooperation among the Ministry of Home Affairs, Ministry of Health, Family Welfare Movement (*Pemberdayaan Kesejahteraan Keluarga* or PKK) and PKMD for training and financing. Employing over one million volunteers known as *kaders*, a *posyandu* offers medical care and support often in remote or underserved areas [9]. *Kaders* are selected by village leaders or committees and are trained through *puskesmas* (community health clinics). While *posyandu* primarily addresses child and maternal health, local chapters adapt their messages and care efforts to meet the needs of their local community. Successful *posyandu* are largely the result of credibility and trust placed in the local *kader*, which leads to increased participation and satisfaction with services within the community [10–12].

Indonesia has a long history of vector-borne disease research [3, 13–16], including recent studies highlighting the seasonal prevalence of malaria on West Sumba [17]. However, there are gaps in information around knowledge, attitudes, and practices (KAP) studies which could help decision makers improve the acceptability and sustainability of interventions to control the vector and the disease [18–20]. Understanding the human dimensions of disease control is critical to tailoring programs that are sustainable and locally acceptable within local communities. Because most mosquito control and disease prevention efforts operate through state and local institutions, qualitative studies with local leaders and decision-makers can provide in-depth knowledge about challenges facing local, community-based mosquito control and disease prevention programs.

This study investigates local knowledge, attitudes, and practices of mosquito-borne disease prevention. The research offers insight into mosquito control and malaria prevention through the perspectives and narratives of residents in East Sumba, Indonesia and highlights the role of community health organizations in malaria prevention.

## Methods

### Ethics statement

The study was reviewed by the National Center for Atmospheric Research (NCAR) (IRB#2015–16) and met the criteria for exemption. Universitas Kristen Wira Wacana Sumba and the University of Arizona deferred to NCAR’s Human Subjects Committee. All locally required approvals were obtained by faculty at Universitas Kristen Wira Wacana Sumba. All participants were over 18 years of age and provided recorded verbal consent before participation in FGDs or KIIs.

### Study settings

The study was conducted in East Sumba Island, located in the province of East Nusa Tenggara in Indonesia. Indonesia is the world’s fourth most populous country, spread across over 17,500 islands located in the Indian Ocean in Southeast Asia. A large population geographically dispersed across thousands of islands, high rates of internal migration, socio-economic inequality, and a decentralized government all make combating malaria challenging in Indonesia [4]. With an average elevation of 367 meters, the climate of Indonesia is tropical, characterized by two distinct seasons–wet and dry. The wet season in our study area in East Sumba Island Regency, East Nusa Tenggara is typically November through March with an average monthly rainfall ranging from 147 mm in November to 274 mm in February.

East Sumba is one of four regencies of Sumba Island, Indonesia. Sumba Island has an area of 11,060 square kilometers and nearly 756,000 people. Comparatively, Sumba Island is slightly larger than nearby Bali (5,780 square kilometers); however, it has only one-sixth of the population. Agriculture is at the center of the local economy in East Sumba, typically in the form of subsistence farming or industrial agriculture in sugar cane plantations [21].

**Fig 1:**
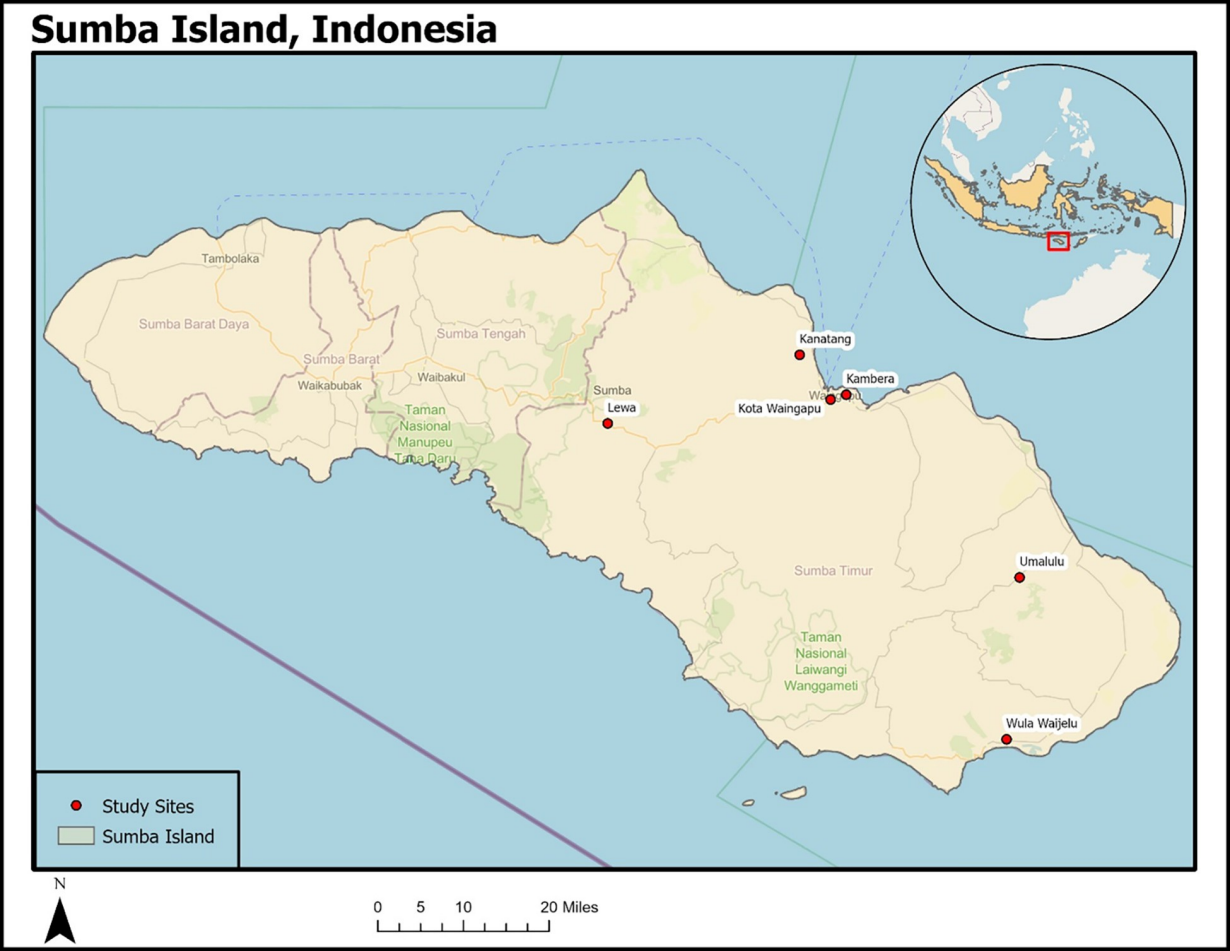
Study area map. Indonesia shapefile obtained from ESRI’s ArcGIS Online.

Subdistricts were stratified by urban/ rural and malaria incidence within the East Sumba Regency, as seen in Fig 1. Six sub-districts that were geographically dispersed were selected to represent a mix of high and low transmission in urban and rural sites (Table 1). Rural subdistricts with relatively low annual malaria incidence (ranging from 22–70 per 1,000 people) were Kambera, Kanatang, and Lewa. Kota Waingapu, the only urban subdistrict, also has low annual malaria incidence (ranging from 39–53 per 1,000 people). Rural subdistricts with high annual malaria incidence (ranging from 166–518 per 1,000 people) were Umalulu and Wula Waijelu (Ministry of Health, Sumba Island, Indonesia).

**Table 1:** Description of subdistricts and annual malaria incidence.

Subdistrict	Urban (U) or Rural (R) & High (H) or Low (L) Incidence	Distance from Kota Waingapu	Cumulative Annual Malaria Incidence		
			2012 incidence	2013 incidence	2014 incidence
**Kota Waingapu**	Urban; low incidence	1 KM	49 per 1000	39 per 1000	53 per 1000
**Kambera**	Rural; low incidence	5 KM	70 per 1000	22 per 1000	34 per 1000
**Kanatang**	Rural; low incidence	6 KM	No Data	No Data	25 per 1000
**Lewa**	Rural; low incidence	60 KM	32 per 1000	22 per 1000	34 per 1000
**Umalulu**	Rural; high incidence	62 KM	259 per 1000	305 per 1000	166 per 1000
**Wula Waijelu**	Rural; high incidence	123 KM	327 per 1000	429 per 1000	518 per 1000

Data collection was conducted from 11–19 November 2015. Malaria incidence in the six selected subdistricts, as well as their distance from Kota Waingapu, the largest town and capital of the province, can be found in Table 1.

### Data collection and analysis

Purposive sampling was used to recruit participants for both Key Informant Interviews (KIIs) and Focus Group Discussions (FGDs). Participants of FGDs and KIIs were recruited with assistance from research collaborators at Universitas Kristen Wira Wacana Sumba in Kota Waingapu, East Sumba, Indonesia. The study was reviewed by the National Center for Atmospheric Research (IRB#2015–16) and met the criteria for exemption. All participants were over 18 years of age and verbal consent was recorded before participation in FGDs or KIIs. The KII participants from the six subdistricts were women and men who held leadership positions within their communities or those who worked in health-related fields (e.g., *kader*, nurse, midwife, pastor/preacher. Most participants of FGDs were women at least 18 years of age (one FGD was comprised of men) who had resided within the subdistricts for at least one calendar year prior to the study. Data for both KIIs and FGDs were collected using semi-structured interview guides [see S1 Text]. FGD and KII were conducted by local collaborators in Sumbanese. A translator was on-site during all interviews and discussions, which allowed the research personnel to take notes in both English and Sumbanese. This facilitated non-Sumbanese researchers to ask specific follow-up questions if needed. All FGDs and KIIs were digitally recorded. Data collection of KIIs and FGDs occurred in person, and within the subdistricts.

Local bilingual research team members transcribed and translated FGDs and KIIs into English. From there, the US-based research team coded the transcripts based on *a priori* themes, including use of ITNs. Emergent themes such as the role of *posyandu* were also identified and analyzed. From this analysis, three major themes were identified: 1) household level prevention and control; 2) community-based prevention and control; and 3) women’s empowerment. This provided an opportunity to gather nuanced information about *a priori* and emergent themes and use the data to inform development of a quantitative household survey which was conducted the following year. A consolidated criteria for reporting qualitative research (COREQ) checklist was utilized to report all criteria necessary [22].

While data collection spanned multiple subdistricts in East Sumba, Indonesia, this study did not set out to collect data that would be generalizable. This data serves to provide in-depth information about the knowledge, attitudes, and practices of local residents in one of the remaining high transmission areas in Indonesia.

## Results

Site specific information from FGDs and KIIs provided data on the knowledge, attitudes, and practices of mosquito control in East Sumba, Indonesia among participants from religious institutions, multiple levels of government, and community-based health programs, including *kaders* and members of the *posyandu* (Tables 2 and 3). The role of *posyandu* in mosquito control was not an initial primary focus of this research; however, after data analysis, its value for communities and stakeholders became evident. Additionally, data revealed that *kaders* working with *posyandu* members were instrumental in combatting malaria at the local level and often not formally recognized for the important role they played in mosquito control and malaria prevention in the regency.

**Table 2:** Key informant interview participants.

Occupation	Gender	Subdistrict
Health Department	Male	Kota Waingapu
District Leader	Male	Umalulu
Local Woman Leader	Female	Umalulu
Nurse	Female	Wula Waijelu
District Staff	Female	Wula Waijelu
Minister/Faith Leader	Male	Wula Waijelu
Minister/Faith Leader	Female	Kota Waingapu
Midwife	Female	Kanatang
Nurse	Female	Kanatang
District Leader	Male	Kanatang
Family Welfare Guidance Prog. (PKK)	Female	Lewa
Legislator	Female	Kota Waingapu
Local Community Leader	Male	Kambera
Head of Senior School	Female	Kota Waingapu

**Table 3:** Focus group discussion participants.

Subdistrict	Number of participants	Occupation(s)
Kambera	7	Homemakers
Kota Waingapu	4	Homemaker/Employed outside the home (e.g., health department and Women’s Empowerment & Family Planning)
Wula Waijelu	7	Homemaker/Employed outside the home (e.g., *kaders*, teachers, minister, and government official)
Umalulu	8	Homemaker/community leaders
Kanatang	5	Homemaker/Employed outside the home (e.g., *kaders)*
Lewa	7	Homemaker/Employed outside the home (e.g., *kaders* and deacons)
Kota Waingapu	7	Men from Kampung Raja

### Household level prevention and control

The distribution of ITNs by the government and its global health partners (e.g., UNICEF, WHO) to at-risk communities to prevent malaria has been shown to decrease malaria prevalence in East Nusa Tenggara [23]. Results from this research showed a consensus among study participants that ITNs were perceived to be effective in preventing malaria and were considered acceptable; this perception echoes results from a previous study in Indonesia that indicate appreciation for free bed net distributions [24]. In Lewa (R/L), one study participant noted that, “before that [distribution of bed nets in 2014] we already use mosquito bed net.” Accounts such as, “The bed net is the top. We rarely use the spray or the mosquito coils” (Wula Waijelu, R/H) and “It is safe when you use mosquito bed nets. That is the most effective way of preventing malaria. You can use it in the morning, day, and night” (Kambera, R/L) demonstrate the perceived effectiveness and importance of ITNs. However, before ITNs were distributed by the government (and their partners), ITN usage was less uniform. Based on information provided by some study participants, ITN use was less homogenous before the government (and its partners) provided them to all households within the community. “In past, the mothers they sewed the bed nets by their own hands” (Kota Waingapu, U/L).

While the government conducts the distribution of ITNs, places of worship partner in the dissemination of information related to the importance of mosquito control products such as ITNs. A faith leader from Wula Waijelu (R/H) said he is regularly invited to health-related meetings, including meetings about malaria. “The meetings (about malaria) are in the health center. It is about the understanding of the community, the importance of using bed nets, and a healthy lifestyle at home, like closing the places of mosquitoes where they lay eggs and the water puddles. That we often speak about through the pulpit at Sunday service at the church.” Another faith leader from Kota Waingapu (U/L) mentioned similar experiences of using church-related activities (e.g., conversations after services, household bible studies) to deliver information to her congregation about health and wellness. And while the faith leader from Kota Waingapu (U/L) mentioned malaria, she referred to chronic illnesses such as diabetes as more commonplace ailments in her congregation. The faith leader went on to say, “Church can be involved because it is quite open if there is something useful for the congregation.” Study participants identified as Christian (79%), Traditional/Marapu (16.4%), Muslim (4%), and Other (0.7%). Throughout the subdistricts, local governments cooperate with churches and mosques, as well as *puskesmas* (community health clinics overseen by the Ministry of Health) and *posyandu*, to provide information about the distribution and use of ITNs as mentioned by FGD participants in Kanatang (R/L) and Wula Waijelu (R/H). “When we want to distribute the mosquito bed nets from *puskesmas*, we announce it through Sunday service in church. We also announce it in the mosque” (Kanatang, R/L).

While ITNs and other products are distributed free of charge to Indonesians, it is unclear how frequently they are used. Several FGD participants stated that ITNs can be uncomfortable and cited factors such as claustrophobia, smell, or feeling too hot once underneath the bed net to use it. For example, one participant from Wula Waijelu (R/H) said, “we live near the beach, perhaps because of that, close to the beach, it is hot, so we sit outside or to other places.” Another woman, also from Wula Waijelu (R/H), added “It is hot, so we sleep outside. The men open their shirts.” While other FGD participants suggested otherwise, such as in Kota Waingapu (U/L), one participant noted that “although it is an extra-large bed, I still use the net. I prefer to feel the heat than to get bitten by the mosquitoes.”

In some cases, certain members of the household will use an ITN while others do not, creating a conversation about gender dynamics and duties in the household. When asked about the discussions related to who uses ITNs in the event of limited resources, as well as who is consulted about the use (e.g., husband, grandparents), the study participants said those are their own decisions. “As a mother, we play an active role to prevent [mosquito-borne diseases]” (Lewa, R/L). In the same discussion another participant added that decisions related to health and disease prevention “dominates by women” (Lewa, R/L). Study participants were also asked to specify who in their household uses an ITN. One study participant replied, “the children, our relatives who stay with us… who use the mosquito nets. For others, we use the nets that we bought” (Wula Waijelu, R/H).

Other forms of mosquito control were also distributed in East Sumba to reduce immature mosquitoes. In addition to ITNs, *Abate* (temephos, an organophosphate larvicide) is distributed across several subdistricts. In Lewa (R/L), for example, “The hospital distributes *Abate* for the mosquitoes…. also, some chlorine to pour it into a well.” The perceived effectiveness of the distribution of *Abate* is found in testimonials about the decrease in number of malaria cases, “We also get *Abate* from *posyandu* which can be used for two weeks. They distribute it every year,” said one participant among a group of women in Kota Waingapu (U/L). Study participants from Wula Waijelu (R/H) expressed some concerns about the effectiveness of other chemical controls; one study participant in Wula Waijelu (R/H) indicated, “It [insecticide] is not too effective because for us who live in an open house on stilts, if we spray, it is spread everywhere. The only thing we do is to buy the mosquito coils.”

Both KII and FGD participants in multiple subdistricts noted that many traditional methods were used to control mosquitoes in the home. “For us, we use the papaya leaves. Sometimes we take the leaves and put them under the bed. We can also use the breadfruit leaves or its fruit which has long flower. It is very good (to chase away the mosquito); we only have to burn one fruit and it will work like a mosquito repellent until the next morning” (Kota Waingapu, U/L). Multiple participants said that consuming papaya leaves was a traditional method to treat malaria. “…take traditional medicine…that’s all I do, there is we have Nimba (Neem) small leaves, 5 sirih (Betel) leaves…and the third the youngest papaya leaves on the top…that just take one…boil all the leaves and drink. I left the medicines from hospital (didn’t drink), I tried that, I tried for 3 days, until today I haven’t got malaria and after that never get the malaria” (Umalulu, R/H). Study participants from Kota Waingapu (U/L) discussed their use of commercial products such as *Nona Mas*, *Autan*, *Soffell* and telon oil. “The Nona Mas oil has the aroma of citronella. It is just like what you said to put the citronela under the bed. I rub the Nona Mas oil on (the children’s) feet and hands when they sleep” (Kota Waingapu, U/L). Another participant in the group noted that “Sometimes, if we want to put the oil on the tray and swing it around to catch the mosquito. They will get sticked and die” (Kota-Waingapu U/L).

### Community-based prevention and control–*Posyandu* and *Kaders*

*Posyandu* were established to achieve health equity in Indonesia and in remote areas are the first line of defense against disease, malnutrition, and other ailments. This community-based health service became an opportunity to engage volunteers, particularly women, to be involved in public health. According to participants across the subdistricts, a typical *posyandu* operates with five *kaders–*similar to what was reported in a 2014 study by Shelley et al. *Kaders* are primarily women, and each volunteer works with approximately 100 children under the age of five [9]. *Kaders* monitor children’s growth, nutritional information, immunization history and record all such health measures through mother-infant *Kartu Menuju Sehat KMS* (Card to Health) cards. *Kaders* also offer basic care to children and promote their development as well as providing parental education. Some FGD participants, who have volunteered as *kaders* at *posyandu* service centers, have access to a small fund that is distributed among *kaders* for their work. “Thus, the government gives a little fund to *posyandu*. It is said to be the support for *posyandu*, but it is not, it is to be distributed routinely among the *kaders*. There is another… as *posyandu* operational fund” (Kota Waingapu, U/L). Evidence suggests that paying volunteers will help retain *kaders* and improve *posyandu* performance [25].

Regular duties of *kaders* are to complete checkups of patients at *posyandu* (i.e., weighing, nutritional advising, counseling), as well as make household visits when parents do not attend routine healthcare visits at the clinic. Visits to the clinic are centered around the care of children under the age of five. While child and maternal health is much of the work done at *posyandu*, their roles can be more expansive, depending on the location. One study participant in Wula Waijelu (R/H) highlighted the importance of door-to-door visits as, “After the D-Day [day of *posyandu*], if there were parents who did not come to *posyandu* (clinic) on the previous day, then we can visit them door to door, to give motivation so they are willing to come in the following month.” The role of *kaders*, particularly in the context of malaria prevention, is absent from much of the literature in Indonesia. Our research in East Sumba has found that *kaders* working at *posyandu*, have taken direct action to control mosquitoes and prevent malaria.

In Wula Waijelu (R/H), one of the participants claimed that malaria was the focus of the *posyandu* where she volunteered. “We talk about malaria; we still have malaria here because the area is close to the forest. The people’s intelligence is still too low and not all people have the activity to clean up the village. And on the mountains, people are spread everywhere.” This account was corroborated by other FGD participants who expressed a concern for environmental conditions or current human behavior that might promote malaria transmission within the community. These comments suggest that posyandu have adjusted their priorities to best serve the communities where they operate.

Organizations such as PKK also participate in mosquito control activities such as suggesting the removal of hanging clothes to keep mosquitoes from harboring around houses. “In the past, there was a program called *dasawisma* through PKK, we even did it at the provincial level. They [PKK] went door to door, they even took down the clothes that were hung. They were great because they were not afraid. They show real action. They gave example how to collect all the dirty clothes” (Wula Waijelu, R/H).

### Women’s empowerment

While women’s empowerment was not the initial or primary focus of *posyandu*, participation in *posyandu* is a way to empower women according to a faith leader from Wula Waijelu (R/H).

“The health department, especially the nearest health center, is really proactive. They really push it [*posyandu*] and facilitate and empower the women. So, the secret here is that if there is continuous guidance to the group, then any group, including a group for women, especially in the area of health, will run well. That’s what I see. But if, after the group has been formed, there is no guidance, no empowerment, then it will just disappear, leaving the name behind.”

The key informant has attended government events on the topic of empowerment and notes that women are the focus of the meetings. On the topic of empowerment, the key informant shared an idea with the Women’s Empowerment Agency Sumba Timur regency to hold a certain number of elected positions in the community for women and also spoke of empowerment regarding women’s participation in community programs and activities.

A focus group participant from Kanatang (R/L) shared, “By being a *posyandu kader*, there are some benefits I get. We learn how to serve, serve the community…but we serve the community with heart because the babies, children, and even the pregnant women around us is a part family.” Empowerment, knowledge, and a sense of community were themes that arose during data collection. One woman from Wula Waijelu (R/H) said,

“For me, yeah…if we get involved in a community, as mentioned by another study participant, of course it can improve our knowledge, and as social creature, we become more, what is it…we have more empathy. So, it is about the sense of humanity toward others; if we can give or do something for other people, it is a happiness for me personally. So, as it is said, we participate in those communities and then we become very busy, it is not for our own family, but we can also share or interact with other people. This life will be more useful and meaningful,” while another added,

“But by participating in those communities that you mentioned before, we can increase our knowledge, and there are spaces that we can enter, for example, in the programs intended for the village, in the programs for the district, I think those are the spaces that we can enter which of course, sorry to say, can give us a reward” (Wula Waijelu, R/H).

When asked about involving women in mosquito control practices, participants were open and interested in the idea. Participants in Wula Waijelu (R/H) expressed a variety of opinions regarding women in mosquito control jobs. One focus group discussant claimed her positionality as a mother and woman would impact her approach to mosquito control saying, “If it is me [fogging,] I am motherly because I have many children and we need to socialize and speak about a few things.” Another added that, “While the men are rude [when they get] out, they take all their instruments.”

## Discussion

### Household level prevention and control

Insecticide treated nets are recognized as an important strategy for preventing transmission of malaria, [26] and several study participants recognizing the importance of ITN use to effectively control malaria, purchased ITNs rather than wait for ITNs to be distributed by the health department at no cost. Purchasing ITNs prior to or in addition to receiving them from the government was found to be commonplace among focus group discussants. This finding is similar to that of a previous study in Java, Indonesia [27].

Most participants noted the widespread use and effectiveness of ITNs, yet some study participants admitted to not always using their ITNs. These findings mirror results from other studies in Southeast Asia where heat was the most oft-cited reason why study participants did not use ITNs [28–31]. Perspectives from FGD participants revealed information related to ITN usage and environmental hazards (such as heat or proximity to bodies of water) in East Sumba which suggests an opportunity to work with communities to identify means to mitigate these risks.

Overall, respondents across all subdistricts found ITNs to be an effective mechanism to control mosquitoes and prevent malaria. While many participants suggested that ITNs may not always be comfortable, they are still important.

Findings from previous studies have indicated that there are several limitations to using top-down approaches to ITN distribution, especially when distribution is not paired with education. For example, top-down approaches in Cameroon and Tanzania found that government distribution of ITNs is not always successful as ITNs have been repurposed for other activities such as fishing [32, 33], indicating that this strategy might not be a sustainable solution. The distribution of ITNs through national initiatives are commonplace yet have mixed results. For example, research from Kenya found that larger households were less likely to receive enough ITNs to cover all household members [34]. While pregnant women and infants are to receive ITNs, some areas are left with too many or too few [35]. Several scholars note that a more localized approach or using extension services and global health partners (e.g., NGOs) would be more sustainable [31, 35]. In East Sumba Island, Indonesia, ITN distribution occurs through the multiple levels of the government as part of a national initiative, distributed through local institutions, like *posyandu*, as well as through global health partners, (e.g., UNICEF). While ITNs are freely available from the government during area-wide distribution events, many households purchase their own either to meet their household demand or due to delays related to distribution.

Mosquito control measures used as alternatives to ITNs, or in conjunction with them, emerged during the data collection process. Commercial products (e.g., *Nona Mas*, *Autan*, *Soffell* and telon oil) as well as locally sourced plants (e.g., papaya leaves, Neem leaves, betel leaves) were discussed by participants regarding their use and perceived efficacy. Papaya leaves have been studied for their efficacy repelling mosquitoes, making them a potential alternative to inorganic ingredients [36]. Additionally, Ahmad et al. 2011 found the consumption of *Carica papaya* leaf extract to be useful in combatting dengue fever although this finding needs further study [37].

### Community-based prevention and control–*Posyandu* and *Kaders*

*Posyandu* seek to serve pregnant women and children under the age of five who are most vulnerable to malaria [8]. Although the original mission of the *posyandu* is child and maternal health, this research has shown that the organization has a wider scope than its original focus. While other study participants confirmed the distribution of mosquito control related products, the timing of distribution varied across study areas; one study participant mentioned that *Abate* is no longer freely distributed in their area.

Future research should pursue a more rigorous examination of the involvement of *posyandu* and *kaders* in mosquito control and malaria prevention in East Sumba. While some information about their work in this context emerged from participants’ replies, explicit questions about the level of training and uniformity of involvement in mosquito control across *posyandu* and among *kaders* were not asked in the study. Further, more attention could be paid to the timing and distribution of the mosquito control measures such as ITNs and temephos.

### Women’s empowerment

In East Sumba, the Family Welfare Empowerment program (PKK), a women’s empowerment group that operates from the national to the neighborhood level, is also involved in the mosquito control in the form of prevention [38]. Taking down (wet) hanging clothes outside was mentioned by several participants across multiple subdistricts as a form of mosquito control, similar to the findings of a qualitative study conducted in Burkina Faso [39]. According to participants of the FGDs in East Sumba, wet clothes are perceived as a space for mosquitoes to breed; however, evidence exists from Gambia that this is more likely a place for mosquitoes to rest [40].

While fogging is a practice used mainly to control *Aedes* mosquitoes and combat diseases such as dengue, women are familiar with these activities and have expressed their interest in these roles. The participants’ perceptions indicate that women might be more appropriate for household visits and may be kinder in the process. A lack of recruitment and training were the chief reasons women have not yet been involved in mosquito control spraying according to women in the study who were interested in participating in vector control [41].

As some participants mentioned, oftentimes domestic duties (and other cultural norms), prevent women from seeking work in vector control. Increased efforts to recruit and educate women about career opportunities in vector control improve gender equity in the workplace and will also combat mosquito-borne diseases [42]. As found in another study, women’s influence, and decision-making power in domestic spaces, makes their involvement an important component of vector control strategies [43].

### Study limitations

Because our original research focus aimed at investigating women in vector control, our data collection process engaged primarily women. Most FGDs and KIIs were mainly women to probe the barriers and opportunities for women to access jobs in mosquito control. Therefore, purposive sampling for this study was undertaken across subdistricts to focus on the role of women. Data collected from semi-structured interviews and FGDs were designed to provide insight into the KAP of participants as they relate to mosquito control and malaria prevention.

## Conclusion

A key finding of this research is the role of *posyandu* in mosquito control and malaria prevention. Expanding or shifting the focus of *posyandu* seems to already be occurring in certain malaria endemic communities, especially in areas where the rates of community infection dictate the need for greater involvement.

The aims of this research were to gain insights into local knowledge, attitudes and practices related to mosquito control and prevention of malaria. Participants of FGDs and KIIs contributed to this research which provided community perspectives and accounts of the current state of malaria control and prevention in East Sumba, setting the stage for future community-led engagement with a goal of malaria elimination.

As malaria cases resurge in Indonesia, more resources and newer technology have been deployed to curb the local disease burden. Community engagement and participation is critical for reaching long-term goals related to mosquito control and malaria prevention. Findings from this study will aid in accomplishing Indonesia’s goal as well as the Global Technical Strategy for Malaria 2016–2030 goal of a 90% reduction in malaria disease burden (from a 2015 baseline) by highlighting issues related to ITNs as well as identifying areas where *posyandu* (and similar institutions) can have a greater impact in vector control.

## Supporting information

S1 File Sumba Island. (ZIP)

S1 Text Interview guide. (DOCX)
