# Supplementary material for: Knowledge, attitudes, and practices of Anopheles mosquito control through insecticide treated nets and community-based health programs to prevent malaria in East Sumba Island, Indonesia
Source: PLOS Glob Public Health. 2022 Sep 2;2(9):e0000241. doi: 10.1371/journal.pgph.0000241 (PMC10021134; doi:10.1371/journal.pgph.0000241)
Supplement: S1 Text — (DOCX) [file pgph.0000241.s002.docx]

**S1 Text: Additional file 1**

**Focus Group Interview Guide**

| **Focus Group Guide**  Accelerating to Equal–Women in Vector Control  Focus Group Guide for Sumba Island, Indonesia:  The main goals of the project are to understand barriers and opportunities for women in vector control and to identify potential strategies for accelerating involvement of women in sustained support for malaria control interventions at multiple levels and sectors.  The purpose of the focus group discussions is to understand current attitudes and practices regarding health concerns, in general and malaria and mosquito control, in particular and to identify barriers to equalizing participation in vector control implementation. Women’s roles in vector control may vary from the household level to the community level to a broader national and international level. These focus groups will provide qualitative data that are intended to guide the development and contextualization of quantitative household level surveys to be conducted in both sites.  **Opening Questions**  1. What activities do women in your community participate in?  Prompt:  a. women’s groups  b. micro-credit orgs  c. agricultural production groups  d. other social networks  e. income generating activities  f. any other activities  2. How do women decide which activities to participate in?  3. What opportunities are there for women to work to improve their community?  a. Are these volunteer positions?  b. Are these paid positions?  c. How are women selected for these positions?  4. As a leader in your community, what steps did you have to take to become a leader?  5. What problems did you encounter?  **Decision Making**  6. What are the barriers to women’s participation in work or community-related activities?  a. gender roles  b. time  c. children  d. household responsibilities  f. lack of education    7. What factors allow some women to have more authority to make decisions in their households?  a. age  b. education  c. socio-cultural  d. religious  e. independent income  8. Tell us about a time when you participated in an event to improve your community.  9. Do you think access to medical care is equal for everyone in your community?  **Health Concerns**  10. What is the primary health concern in your community?  11. Are the concerns different for adults versus children?  12. How big a concern is malaria for your community?  13. Have you been involved in malaria control in your community?  14. From what you’ve learned, how do people in your community think they get malaria?  15. What are some things that women could do at a household level and at a community level to reduce the burden of malaria in your community?  16. What would be the role of your organization in facilitating the involvement of women in mosquito control?  **Risk Factors**  17. Are there community level changes to the local environment that would reduce the burden of malaria in your community?  Prompt:  a. clearing vegetation  b. draining ditches  c. organizing community clean-ups  d. other?  18. What are the key community level messages that you think are important for malaria prevention?    19. Are there certain families in the community who seem to rarely/never have malaria? Why?  **Malaria prevention**  20. What are the strategies used in your community to reduce malaria?  a. bed nets  What role do women have in distributing bed nets? Does anyone provide education at the time of distribution about best practices for hanging, cleaning, maintaining?  b. fogging  What role do women play in fogging? Are there problems with fogging? What role could women play in improving the uptake of fogging?  21. If there were a new malaria prevention product, what would the role of women be in  Prompts:  a. The distribution of the product  b. The education the community about the product?  c. What would be your first steps to involve women?  **Here is an example of a spatial repellent that is being tested in West Sumba. This product is currently for test purposes only and is not for sale. It can be used to keep mosquitoes out of a 3 square meter space for about 2-4 weeks. It can be hung on the wall using a stapler, thumb tack, double-sided tape or a nail.**  22. How well do you think the community would accept this product?  **Access to Information**  23. What is the best way to provide the community with information?  Prompt:  a. community health workers  b. churches  c. schools  d. village elders  e. informal networking such as borehole gatherings/gossip  f. informal networking such as markets  g. other informal networks? |
| --- |
